# Valorization of Cork Stoppers, Coffee-Grounds and Walnut Shells in the Development and Characterization of Pectin-Based Composite Films: Physical, Barrier, Antioxidant, Genotoxic, and Biodegradation Properties

**DOI:** 10.3390/polym16081053

**Published:** 2024-04-11

**Authors:** Rui M. S. Cruz, Bernhard Rainer, Isabella Wagner, Victoria Krauter, Magda Janalíková, António A. Vicente, Jorge M. Vieira

**Affiliations:** 1 Department of Food Engineering, Institute of Engineering, Universidade do Algarve, Campus da Penha, 8005-139 Faro, Portugal; 2 MED—Mediterranean Institute for Agriculture, Environment and Development & CHANGE—Global Change and Sustainability Institute, Faculty of Sciences and Technology, Campus de Gambelas, Universidade do Algarve, 8005-139 Faro, Portugal; 3 Packaging and Resource Management, Department Applied Life Sciences, FH Campus Wien, University of Applied Sciences, 1100 Vienna, Austria; bernhard.rainer@fh-campuswien.ac.at (B.R.); isabella.wagner@fh-campuswien.ac.at (I.W.); victoria.krauter@fh-campuswien.ac.at (V.K.); 4 Department of Environmental Protection Engineering, Faculty of Technology, Tomas Bata University in Zlín, Vavrečkova 275, 760 01 Zlín, Czech Republic; mjanalikova@utb.cz; 5 CEB—Centre of Biological Engineering, Campus de Gualtar, University of Minho, 4710-057 Braga, Portugal; avicente@deb.uminho.pt (A.A.V.); jorgevieirabcd@gmail.com (J.M.V.); 6 LABBELS—Associate Laboratory, 4710-057 Braga, Portugal

**Keywords:** biodegradable, food packaging, pectin film, physico-mechanical, waste valorization, sustainability

## Abstract

The development of sustainable materials from the valorization of waste is a good alternative to reducing the negative environmental impact of plastic packaging. The objectives of this study were to develop and characterize pectin-based composite films incorporated with cork or cork with either coffee grounds or walnut shells, as well as to test the films’ genotoxicity, antioxidant properties, and biodegradation capacity in soil and seawater. The addition of cork, coffee grounds, or walnut shells modified the films’ characteristics. The results showed that those films were thicker (0.487 ± 0.014 mm to 0.572 ± 0.014 mm), more opaque (around 100%), darker (L* = 25.30 ± 0.78 to 33.93 ± 0.84), and had a higher total phenolic content (3.17 ± 0.01 mg GA/g to 4.24 ± 0.02 mg GA/g). On the other hand, the films incorporated only with cork showed higher values of elongation at break (32.24 ± 1.88% to 36.30 ± 3.25%) but lower tensile strength (0.91 ± 0.19 MPa to 1.09 ± 0.08 MPa). All the films presented more heterogeneous and rougher microstructures than the pectin film. This study also revealed that the developed films do not contain DNA-reactive substances and that they are biodegradable in soil and seawater. These positive properties could subsequently make the developed films an interesting eco-friendly food packaging solution that contributes to the valorization of organic waste and by-products, thus promoting the circular economy and reducing the environmental impact of plastic materials.

## 1. Introduction

Plastic is a widely used material in the (food) packaging sector. To date, petroleum-based polymers such as polyethylene (PE), polypropylene (PP), polyethylene terephthalate (PET), and polystyrene (PS), as well as a few other materials, dominate this area of application [1]. This is attributable to the various advantages of this material group (e.g., barrier properties, mechanical–physical strength, chemical resistance, moldability, sealability, lightness, low cost) [2,3]. However, mismanaged plastic waste streams exhibit an enormous negative impact on the environment due to their low degradation rate and the accumulation of considerable amounts of plastic material in the environment. This includes the presence of micro- and nano-plastic particles, which endanger different ecosystems on land and at sea [4,5].

To promote a sustainable future, different alternatives and strategies have been developed and applied by governmental bodies, the industry itself, and the scientific community, mainly in North America and Europe, in recent years. These include banning single-use plastics and introducing new recycling systems and new plastic packaging derived from recycled material, which contribute to the circular economy [6,7]. Moreover, bio-based and/or biodegradable materials have come into the spotlight [8,9]. In this context, different studies have been reported that use polysaccharides (e.g., starch, cellulose, pectin, alginate, and chitosan) to develop new edible and non-edible films for the food industry [10] and potentially more environmentally sustainable alternatives that contribute to drastically reducing the environmental pollution caused by plastic materials [7].

Cork is a very interesting material, which is mainly composed of suberin and lignin and has great properties, such as lightness and flexibility [11]. In the food industry, cork is mainly used as a cork stopper for wine and spirit bottles. After consumption, although used cork stoppers are recyclable and some initiatives collect, recycle, and generate new products, the largest fraction of cork stoppers is considered waste and is simply discarded [12,13]. In a study reported by Ihamouchen et al. [14], cork waste was combined with polycaprolactone to develop a new cork-based material. More recently, Moutinho et al. [15] presented a study in which bio-based expanded cork polymer composites with poly(lactic acid) were developed.

Relevant by-product flows come also from coffee production and processing. In particular, coffee grounds are the main by-product produced after coffee brewing [16,17]. Coffee grounds are rich in polysaccharides, including cellulose, galactomannans, and arabinogalactans [18]. Recently, Cobo-Ceacero et al. [16] used coffee grounds with clays, among other organic wastes, to obtain lightweight expanded aggregates. In another study, Cataldo et al. [19] used coffee grounds as a filler with pectin to prepare biocomposite films. Moustafa et al. [20] developed and characterized bio-based poly(butylene adipate-co-terephthalate) (PBAT) composites filled with coffee grounds bio-additives.

Another example is the nut shells generated from walnut processing. This by-product has high strength and is composed mainly of lignin, followed by cellulose, hemicellulose, and finally ash. It can be incinerated or used as a raw material in different industrial processes, such as flours and bases for soaps [16,21]. More recently, Beskopylny et al. [22] used walnut shells as an additive in the production of concrete. In another study, Sowińska-Baranowska et al. [23] showed the potential application of walnut shells as biofillers for natural rubber composites. Girgin and Tugrul [24] presented a study in which an edible film was developed with dry walnut shells and plasticized with glycerol and/or sorbitol.

These types of waste exist in large amounts (e.g., tons per day of coffee grounds), and their reuse and valorization in the development of new end-use products and materials, such as (food) packaging, can be a sustainable alternative to contribute to the circular economy and reduce the environmental burden caused by mismanaged plastic materials [25]. However, the actual sustainability of the solutions developed must always be considered and evaluated on a case-by-case basis [26].

To our knowledge, no study is available in the literature that reports the use of cork stoppers with coffee grounds or walnut shells in the development of pectin-based composite packaging films. Thus, the objectives of this study were to develop and characterize pectin-based composite films incorporated with cork and cork with coffee grounds or walnut shells, as well as to test the films’ genotoxicity, antioxidant properties, and biodegradation capacity in soil and seawater. This allows us to take a first step toward the valorization of these side streams.

## 2. Materials and Methods

### 2.1. Materials

Apple pectin (50–70% degree of esterification; MW = 60,000–130,000 g/mol) was supplied by Sigma (Algés, Portugal). Glycerol and NaBr were purchased from JMGS (Odivelas, Portugal). Cork stoppers (ground to a particle size < 63 μm; moisture content = 4%; suberin (33–50%), lignin (20–25%), cellulose and hemicelluloses (12–20%) [15]) were kindly supplied from local restaurants in Faro, Portugal. The grounds from Arabica and Robusta coffee beans (ground to a particle size < 63 μm; moisture content = 6%; cellulose (16–50%), hemicelluloses (17–33%), lipids (13–15%) [18]) were kindly supplied by the ISE-UAlg bar (Faro, Portugal), and the walnuts from the variety Chandler (ground to a particle size < 63 μm; moisture content = 5%; lignin (37–50%), cellulose (18–24%), hemicelluloses (22–36%) [21,23]) were bought from a local market (Faro, Portugal). The soil (Eco Grow) was purchased in AKI (Faro, Portugal).

### 2.2. Preparation of the Films

Pectin films (A) were prepared based on the method reported by Mendes et al. [27], with some modifications. Apple pectin (0.5 g) was mixed to 25 mL of water (previously heated to 75 °C) and stirred for 10 min. Then, the film-forming solution was homogenized at 20,500 rpm for 5 min (Ultra-Turrax T25, Janke & Kunkel, Staufen, Germany) and 1.5 mL of glycerol was added. After this period, the film-forming solution was stirred for 1 h at 40 °C. 

The pectin films incorporated with cork only or with cork with coffee grounds or walnut shells were prepared as follows: 1 g of cork (B); 0.75 g of cork (C); 0.75 g of cork plus 0.25 g of coffee grounds (D); and 0.75 g of cork plus 0.25 g of walnut shells (E) (based on preliminary tests to obtain films with a homogeneous appearance; materials ground to a particle size < 63 µm) were added to 25 mL of water (previously heated to 75 °C) and stirred for 10 min. Then, the same method was followed as previously referred to for pectin films. 

Then, the film-forming solutions were degassed for 5 min and poured into Petri dishes to dry for 48 h at 25 °C. After drying, the films were removed from the Petri dishes and were stored before analysis at 25 °C and 57% relative humidity (using a NaBr solution).

### 2.3. Thickness and Water-Vapor Permeability (WVP)

A digital micrometer (No. 293-5, Mitutoyo, Kawasaki, Japan) was used to measure the thickness of each testing sample at different points (3 replicates). Average values were then used to determine the WVP. A standard procedure, with some modifications [28], was used to measure the WVP of the films. A volume of 50 mL of distilled water was added to the permeation cell to generate a 100% RH (2337 Pa vapor pressure at 20 °C), and the film was sealed at the top of the cells. Then, an analytical balance (AE200, Mettler Toledo, Barcelona, Spain) was used to weigh each cell. The cells were placed inside a desiccator with silica (0% RH; 0 Pa water vapor pressure; a fan inside the desiccator was used to maintain the air circulation constant). The analysis was conducted in triplicate, and modifications in the weight of the cells were recorded at intervals of 2 h to measure moisture loss over time until a steady state was reached. The films’ WVP (g m^−1^ s^−1^ Pa^−1^) was determined by the following equation:

(1) WVP=(WVTR×X)/∆P

where WVTR = water-vapor transmission rate (g m^−2^ s^−1^) through the film, calculated from the slope of the curve divided by the film’s area; X = the film’s thickness (m); and ΔP = the partial vapor-pressure difference (Pa) across the two sides of the film.

### 2.4. Color and Opacity

A colorimeter (CR 400, Minolta, Tokyo, Japan) was used to perform color measurements. The colorimeter was previously calibrated with a standard white tile (EU certified; L* = 84.67, a* = −0.55, and b* = 0.68), which recorded the spectrum of reflected light to determine the parameters L*, a*, and b*. The opacity of each film was calculated based on the method reported by Martins et al. [29]. A relationship between the opacity of each film on a black standard (Yb) and the opacity of each film on a white standard (Yw) was considered, as can be seen in the equation:

(2) $$Opacity\;\left(\%\right)=Yb/Yw\times 100$$

Three samples from each film were analyzed, and three measurements were performed on each sample.

### 2.5. Solubility and Moisture Content 

The method reported by Casariego et al. [30] was used to measure the water solubility and moisture content of the films. The film solubility in water was calculated as the percentage of soluble material after 24 h of immersion in water. A 2 cm diameter disk of each film was dried in an oven at 105 °C until constant weight to obtain the initial dry matter of the films (the films′ moisture content was determined at this part of the method). Then, the sample was immersed in 50 mL of deionized water and moderately shaken (20 °C, 24 h). After 24 h, the insolubilized films were first filtered and then dried in an oven (105 °C, 24 h) to determine the weight of the dried matter that was not solubilized in water. Three replicates were obtained for each sample. The water solubility (%) of the films was calculated as follows:

(3) $$Solubility\;\left(\%\right)=(Mi-Mf)/Mi\times 100$$

where Mi is the initial mass and Mf is the final mass of the sample.

### 2.6. Texture Measurements 

According to a standard method [31], a texture analyzer (TA.XT Plus Texture Analyzer, Stable Micro Systems, Godalming, UK) with the software “Exponent version 6.1.16” (Stable Micro Systems, Godalming, UK) was used to determine the mechanical properties of each film. 

The samples were cut into 20 mm wide, 100 mm long strips, which were positioned between tensile grips. The crosshead speed and the initial grip separation were set at 1.0 mm/s and 80 mm, respectively. The elongation at break and the tensile strength (force/initial cross-sectional area) were determined directly from the strength curves vs. elongation curves by using the software “Exponent version 6.1.16”. Young′s modulus (Equation (4)) was calculated as the slope of the initial linear portion of this curve. Six measurements were performed for each sample.

(4) E=σ/ε

where E represents Young’s modulus, σ is the tensile stress (force per unit area), and ε is the axial strain (deformation).

### 2.7. Total Phenolic Content and Antioxidant Activity

Each sample (0.5 g) was mixed with 70% methanol in a ratio of 1:10 (*w*/*v*) on a magnetic stirrer for 1 h at 50 °C. The mixtures were filtered (0.45 µm), and the supernatants were used to determine the total phenolic content (TPC) and antioxidant activity. Briefly, 100 µL of sample extract was mixed with 1 mL of Folin–Ciocalteu reagent and left for 5 min in the dark at room temperature. Next, 1 mL of Na_2_CO_3_ was added, mixed, and left in the dark for 30 min at room temperature. The blank solution was prepared in the same way except for the addition of sample. TPC was measured by a spectrophotometer (Specord 210, Analytik Jena, Jena, Germany) at 765 nm [32]. TPC was calculated as gallic acid equivalents from the calibration curve standard solutions (0 to 1000 mg/L), and the final results were expressed as mg of gallic acid equivalent per gram of film (mg GA/g). 

The antioxidant activity of the samples was determined by quenching the synthetic radicals ABTS^+^ and DPPH. The ABTS radical method was performed with modifications according to Re et al. [33]. Before the analysis, an ABTS stock solution was prepared using 3.5 M ABTS and 60 mmol/L K_2_S_2_O_8_ in a ratio of 1:50. After 16 h incubation at room temperature, the ABTS working solution was prepared by mixing 2.5 mL of ABTS stock solution with 97.5 mL of acetic buffer (pH 4.3). A volume of 50 μL of the sample extract was mixed with 4.0 mL of the ABTS working solution. The depletion of absorbance was measured spectrophotometrically (Specord 210, Analytik Jena, Jena, Germany) at 734 nm after 30 min of incubation at room temperature.

DPPH radical measurement was performed with modifications according to Ferri et al. [34]. The sample extract (210 µL) was mixed with 4 mL of 1.1 M DPPH radical solution dissolved in MeOH, incubated for 1 h at room temperature, and the absorbance was measured at 515 nm (Specord 210, Analytik Jena, Jena, Germany). Trolox (0 to 200 mg/L for the DPPH method and 0 to 400 mg/L for the ABTS method) was applied as a reference standard in both assays. The final results of the determination of antioxidant activity were expressed as mg of Trolox equivalent per gram (mg TE/g).

### 2.8. Fourier-Transform Infrared Spectroscopy (FTIR)

ATR-FTIR was used to obtain information about the interactions between components in the films. A PerkinElmer 16 PC (Boston, MA, USA), with attenuated total reflectance mode (ATR), was used to record the FTIR spectra of each film. For each spectrum, 16 scans at 4 cm^−1^ resolution were used, for a spectral range of 400–4000 cm^−1^. All samples were analyzed at room temperature (20 °C).

### 2.9. Thermogravimetric Analysis (TGA)

A thermogravimetric analyzer (TGA/DSC, Mettler Toledo, Schwerzenbach, Switzerland) was used to determine the thermal stability of each film by recording the weight loss as a function of temperature. Approximately 10 mg of each film was heated to 900 °C (heating rate: 20 °C/min) under a nitrogen gas flow rate of 50 mL/min.

### 2.10. Scanning Electron Microscopy (SEM)

The surface of each film was analyzed using a desktop scanning electron microscope and the software “ProSuite version 3.0”.

Each film was added to aluminum pin stubs with electrically conductive carbon adhesive tape (PELCO Tabs™). Then, the films were coated with 2 nm of Au (20 Å) for improved conductivity. The aluminum pin stub was then placed inside a Phenom Standard Sample Holder (SH) at 5 kV.

### 2.11. Biodegradation Tests

#### 2.11.1. Seawater

Each film was tested according to the method used by Accinelli et al. [35], with some modifications. First, triplicates of each film were cut, with the dimensions 3 cm × 2 cm, and submerged in 300 mL of seawater (pH = 7.20). Then, the samples were shaken at 150 rpm (KL2 shaker, Edmund Bühler, Tübingen, Germany) and 25 °C. The films′ appearance was photographed during the time of the analysis. 

#### 2.11.2. Soil

Triplicates of each film were cut (3 cm × 2 cm) and placed inside a polyethylene net (5 cm × 4 cm; mesh opening 4 mm) using the method reported by Altaee et al. [36], with some adaptations. All samples were placed in a rectangular vase (71 cm × 26 cm × 25.5 cm) with the following soil properties: pH = 5.5–6.5; humidity = 50–60%; organic matter ≥ 70%; conductivity = 0.2–1.2 EC; nitrogen = 80–150 mg/L; phosphorus = 80–150 mg/L, and potassium = 80–150 mg/L. The films were placed at a distance of 11 cm from the surface and with a distance of 5 cm between each film. The experiment was performed at 25 °C and used 500 mL of water every 7 days. The films′ appearance was photographed, and the area of biodegradation was measured over the time of the analysis. 

### 2.12. Genotoxicity Test

For each sample (A-E), two solvents were used: 95% ethanol (from 99.9%, LiChrosolv^®^, Merck, Darmstadt, Germany) and 3% acetic acid (from 99% acetic acid, Carl Roth, Karlsruhe, Germany). The samples received the suffix x_A if dissolved in acetic acid or x_E if the extraction was carried out with ethanol. 

#### 2.12.1. Acetic Acid Extraction

Three mL of solvent was added to each sample in a centrifuge tube. Sample A_A immediately formed wrinkles after the solvent was added, and it was dissolved completely after 5 min. The other samples start to dissolve after around 30 min. To accelerate the dissolution process, all samples were put into an ultrasonic bath under the following conditions: 80 kHz, 50 °C, 100% power. After 2 min in the ultrasonic bath, samples B_A and D_A had dissolved as well. After another 2 min in the bath, combined with a prior stirring with a spatula, all samples had dissolved in the acetic acid. Centrifugation took place under the following conditions: 4400 rpm for 4 min. The supernatant was removed and used for further analyses with the Ames MPF test, and the precipitate was discarded after drying. To determine the concentration of the extracted sample in the solvent, the tube was weighed at every step. Sample A_A did not form a precipitate; it completely dissolved in the ethanol. For the other samples, more than 50% dissolved in the acetic acid. The details are presented in Table 1. The solvent (3% acetic acid) was used as a blank value in the subsequent genotoxicity (Ames) test.

#### 2.12.2. Ethanol Extraction

A volume of 3 mL of solvent was added to each sample in a centrifuge tube. The sample was extracted for 24 h at 60 °C. None of the samples dissolved during this time; however, the ethanol used for all the samples other than A_E took on the color of the cork. The samples were put into an ultrasonic bath for 2 min under the following conditions: 80 kHz, 50 °C, 100% power. The ethanol was moved into a sample vial, and the leftover samples were dried at 60 °C to determine the sample concentration in the ethanol. Only 11% of the sample A_E dissolved in the ethanol; for the other samples, it was around 50%. Overall, a sample concentration of 46–57 mg/mL ethanol was obtained for all samples other than A_E (where it was only 1.5 mg/mL). The details are presented in Table 2. The solvent (95% ethanol) was used as the blank in the following genotoxicity (Ames) test.

#### 2.12.3. Ames Test 

In order to test for possible genotoxicity of the samples, the Ames MPF test was carried out with two different test strains: *Salmonella* Typhimurium TA98 for frameshift mutations and TA100 for point mutations. The tester strains were purchased from Xenometrix (Allschwil, Switzerland). The overnight pre-cultures were grown with nutrient broth No. 2 (purchased from Oxoid Ltd., Basingstoke, UK) with 50 µg/mL of ampicillin (Sigma-Aldrich, Steinheim, Germany). The test method is based on the official Xenometrix instructions, with some adaptations [37]. To test for genotoxins that require metabolic activity, 4.5% phenobarbital/β-naphtoflavone-induced rat liver S9 (Xenometrix, Allschwil, Switzerland) with a co-factor mix was used in combination with each test strain. To test for inhibitory properties of the materials on the ability of the tester strain to detect positive effects, each sample was tested with and without a standard positive control substance (2-nitrofluorene, 98%, Sigma Aldrich, Steinheim, Germany, for TA98 -S9; 4-nitroquinoline 1-oxide ≥ 98%, Sigma Aldrich, Steinheim, Germany, for TA100 -S9; and 2-aminoanthracene, 96%, Sigma Aldrich, Steinheim, Germany, for TA98 and TA100 +S9).

For the evaluation of the results, a base line was calculated from the background mutation rate from the solvent control plus one standard deviation. Any sample that yielded a response >2-fold over this baseline was considered positive for mutagenicity.

### 2.13. Statistical Analysis 

The results were expressed as the mean and standard deviation of at least three replicates. The experimental data were analyzed with IBM SPSS (Statistical Product and Service Solutions) version 29. An analysis of variance (ANOVA) was performed to detect significant differences among the samples. The significance level was set at 0.05. The least significant difference (LSD) was used as a post hoc test to detect which pair/pairs of samples presented significant differences.

## 3. Results and Discussion 

### 3.1. Physico-Mechanical and Antioxidant Properties

**Figure 1:**
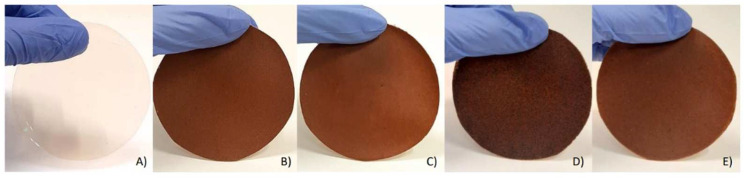
Developed films: (**A**) pectin; (**B**) pectin/1 g cork; (**C**) pectin/0.75 g cork; (**D**) pectin/0.75 g cork/0.25 g coffee grounds; (**E**) pectin/0.75 g cork/0.25 g walnut shells.

Figure 1 presents the developed films. The addition of cork, cork with coffee grounds, or cork with walnut shells to the pectin contributed to the development of opaque and brownish films. All films showed a homogenous surface, except for the film incorporated with coffee grounds, in which a rougher surface was obtained. 

Table 3 shows the physical, mechanical, and antioxidant parameters of the control and the pectin composite films with cork, cork and coffee grounds, or cork with walnut shells. The thickness of the pectin film significantly increased (*p* < 0.05) with the addition of cork, cork and coffee grounds, or cork and walnut shells. Film C was also significantly different (*p* < 0.05) from all the other supplemented films due to its lower solid mass (0.75 g) of cork. The addition of additives to pectin films may contribute to increasing the final thickness of the developed films. In a study reported by Nisar et al. [38], pectin films were enriched with young apple polyphenols. The addition of polyphenols contributed to an increase in the thickness of the films. In another study, Mendes et al. [39] developed pectin films reinforced with spent coffee grounds. The results also showed an increase in thickness after the incorporation of the spent coffee grounds. Mendes et al. [27] also developed pectin films integrated with cocoa butter, and the results showed an increase in the thickness of the pectin films with the incorporation of cocoa butter. Valdespino-León et al. [40] used coffee mucilage to develop biodegradable electrosprayed pectin films. The results are also in agreement with those obtained in this study. In general, the increase in thickness is related to the increase in solid content of the supplemented films.

In terms of color, the pectin film (A) was much lighter and more transparent compared with all the other films. The addition of cork with coffee grounds or walnut shells affected the color and opacity of the pectin composite films, as previously referred to. The L* value of the pectin film significantly decreased (*p* < 0.05) by more than 50% (from 91.86 to values between 25 and 34) after the incorporation of any of the compounds used in this study. Moreover, film D was the one that presented the lowest value (25.30) (*p* < 0.05). This result is mainly related to the dark brown pigments of the coffee grounds, melanoidins, which contribute to darker films [41]. For the a* parameter, the addition of cork, coffee grounds, and/or walnut shells significantly increased (*p* < 0.05) the redness of the films. The results for the b* parameter showed a significant reduction (*p* < 0.05) in the film that incorporated coffee grounds. The yellowness of this film dropped by more than 50% compared with the pectin film. As expected, the opacity of the pectin film increased from 12% to practically 100% after the addition of cork, coffee grounds, or walnut shells. Mendes et al. [39] also reported lower values of lightness (L*) after the addition of coffee grounds to pectin films. The same study also showed a significant increase in the redness of the films with coffee grounds, while the b* parameter increased. Nisar et al. [38] also reported a decrease in the L* values and an increase in the a* and b* values in pectin films enriched with thinned young-apple polyphenols. In another study, Mendes et al. [27] showed that pectin films integrated with cocoa butter also had higher opacity.

The films with cork, cork with coffee grounds or walnut shells showed significantly (*p* < 0.05) higher values of WVP compared with the pectin films. The higher values of WVP may be related, on the one hand, with the higher availability of hydroxyl groups to bind water molecules and, on the other hand, with the greater thickness and more porous structure with lower crystallinity of those films, allowing the passage of gases [40,42,43,44,45]. In a study reported by Valdespino-León et al. [40], the WVP also presented higher values in pectin films incorporated with coffee mucilage. Chen et al. [46] also showed higher values of WVP in pectin-/tara gum-based films with ellagitannins from the unripe fruit of *Rubus chingii* Hu. On the other hand, Ren et al. [47] showed that the addition of calcium propionate and polyvinyl alcohol to pectin produced more compact films, leading to an increase in the water-vapor barrier. In terms of water solubility, the pectin film was fully soluble in water while all the other films presented significantly (*p* < 0.05) lower water solubility. The opposite was observed for the moisture content; the pectin film showed significantly lower values compared with all the other films.

The mechanical properties of the pectin film were affected by the addition of cork, cork with coffee grounds or cork with walnut shells. These films presented a less resistant structure, showing lower values of tensile strength compared with the control film. Moreover, the films incorporated only with cork presented the highest values of elongation at break (32.24 ± 1.88% to 36.30 ± 3.25%). This result may be due to the formation of less dense structures in the films B and C [48]. According to Re et al. [33], the film resistance (tensile strength) and the stretching capacity (elongation at break) is related to the number of intermolecular interactions through H-bonds between the additives and the polymer matrix. Valdespino-León et al. [40] showed similar results for the elongation at break. The pectin films showed lower elongation at break after the addition of coffee mucilage.

The pectin film showed antioxidant properties, which might be related to the presence of hydroxyl groups in the pectin chain [47]. Nevertheless, the antioxidant properties of the pectin composite film were significantly (*p* < 0.05) increased with the addition of cork, coffee grounds, or walnut shells. The free radical scavenging activities (both DPPH and ABTS) as well as the TPC were improved more than sixfold compared with the control films. These results may be attributed to the higher presence of polyphenolic compounds in cork, coffee grounds, and walnut shells. The antioxidant properties of these films can be an interesting feature besides their packaging function in terms of food shelf-life extension. Up to 15 phenolic compounds were identified in cork extracts; in particular, ellagic acid, gallic acid, protocatechuic acid, salicylic acid, naringenin, or quinic acid [49]. About seven bioactive compounds, including caffeine, caffeoylquinic acid, and caffeic acid, were found in coffee grounds [50], and hydrojuglone was the main antioxidant compound in the walnut-shell extract [51]. Our results are in line with the ones reported by Nisar et al. [52], who also showed that the antioxidant properties of citrus pectin films increased after the addition of clove-bud essential oil. Sabbah et al. [53] reported the use of olive and guava leaf extracts in the development of active pectin films. The study showed that the addition of 0.2% (*w*/*v*) of olive leaf extract to the films increased the antioxidant activity compared with the control and with the 0.1% *w*/*v* olive leaf extract. On the other hand, the incorporation of guava leaf extract into the films showed higher antioxidant activity compared with the olive leaf extract at the same concentration. In another study, Oliveira et al. [15] clearly showed the use of phenolic-rich extracts obtained from coffee by-products in the development of active polysaccharide films. Walnut shells are also reported to be rich in active compounds with excellent antiradical activity, such as phenolic acids and ferulic acids [54]. Quilez-Molina et al. [55] reported the development of thermoplastic starch films with the incorporation of walnut shells. The results showed a significant increase in the antioxidant activity of thermoplastic starch films incorporated with 10 wt % of walnut shells. Our study also showed that the pectin composite film incorporated with cork and walnut shells presented the highest values of TPC, DPPH, and ABTS. These properties are interesting since, in the long term, they can have a positive impact on the packaged food and contribute to higher antioxidant protection.

### 3.2. Fourier-Transform Infrared Spectroscopy (FTIR)

**Figure 2:**
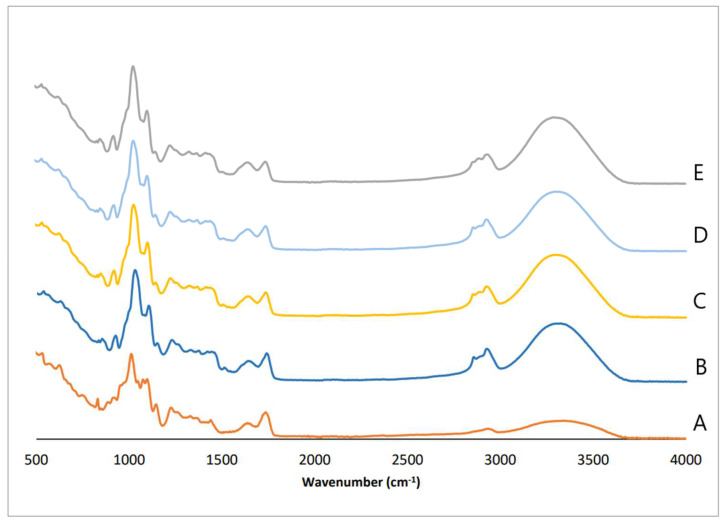
FTIR spectra: A—● pectin; B—● pectin/1 g cork; C—● pectin/0.75 g cork; D—● pectin/0.75 g cork/0.25 g coffee grounds; E—● pectin/0.75 g cork/0.25 g walnut shells.

Figure 2 shows the FTIR spectra of the control film and the films with cork, cork with coffee grounds, or cork with walnut shells. A broad peak ranging from 3700 to 3000 cm^−1^ corresponds to the stretching of O-H because of hydrogen bonding interactions in the galacturonic acid [56]. The peak at 2935 cm^−1^ is attributed to the stretching of C-H bonds [57], and the bands at 1748 and 1626 cm^−1^ are attributed to the absorptions by esterified and free carboxyl groups of pectin, respectively [58,59]. In addition, the bands at 1103 and 1026 cm^−1^ were assigned to C–O–C stretching vibrations of the polymer chain structure [60].

According to Mendes et al. [39], the shoulder-like band at 2935 cm^−1^, related to the stretching vibration of C-H bonds, increased in intensity in the composite films, probably due to single-bond CH_2_ and single-bond CH_3_ groups of the cork, coffee-grounds, and walnut-shell components (such as lignin, hemicellulose, etc.). Moreover, differences between the control film and the composite films in the band associated with the single-bond OH stretching vibration (3700–3000 cm^−1^) may be related to the hydrophilic characteristic loss of the composite films, as confirmed by the permeability analysis.

In general, the addition of these compounds to the pectin composite film exhibited a similar pattern compared with the control film. Their incorporation probably contributed to weaker intermolecular forces between the chains of adjacent macromolecules [61]. This result is corroborated, as previously referred to, by the flexibility shown by the films incorporated with cork or cork with coffee grounds or walnut shells.

### 3.3. Thermogravimetric Analysis (TGA)

**Figure 3:**
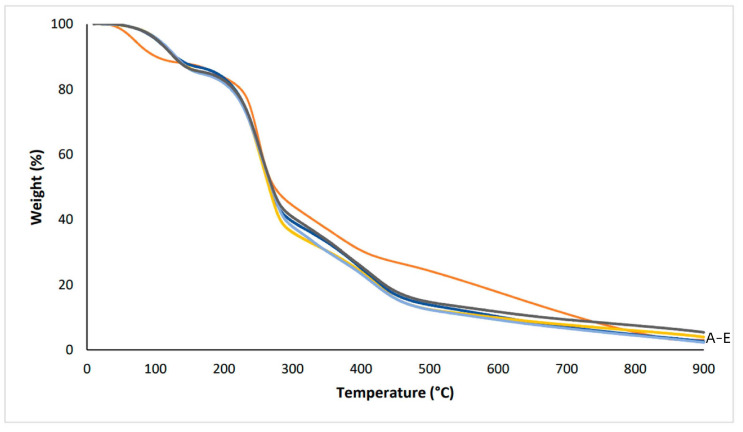
TGA curves: A—● pectin; B—● pectin/1 g cork; C—● pectin/0.75 g cork; D—● pectin/0.75 g cork/0.25 g coffee grounds; E—● pectin/0.75 g cork/0.25 g walnut shells.

Figure 3 presents the TGA curves for the pectin film and the composite films incorporated with cork, cork with coffee grounds, or cork with walnut shells. The thermal stability of the films was evaluated and showed four main defined weight-loss stages. For the pectin film, around 12% and 5% weight losses were observed in the first and second stages in the 10–136 °C and 136–204 °C temperature range, respectively, which corresponds to the water evaporation and low-molecule-mass volatile compounds’ release. The third stage (204–308 °C) corresponds to a higher weight loss (40%) due to polysaccharide depolymerization and decomposition. The last step (308–810 °C) is the thermal decomposition with carbonaceous material formation, presenting a weight loss of 38%. The residue at 810 °C was around 5% [27,47].

The pectin composite films incorporated with cork, coffee grounds, or walnut shells showed similar curves compared with the pectin film. The main weight loss was observed in the stage corresponding to the temperature range between 165 °C and 325 °C, with values of 46–52%. The results showed that the addition of cork or cork with coffee grounds or walnut shells accelerated the degradation of the pectin films.

### 3.4. Scanning Electron Microscopy (SEM)

**Figure 4:**
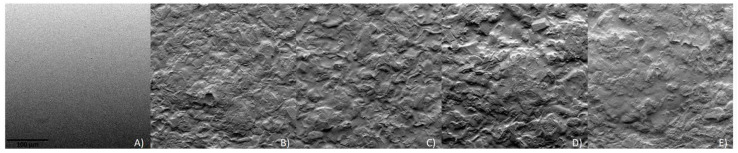
SEM micrographs: (**A**) pectin; (**B**) pectin/1 g cork; (**C**) pectin/0.75 g cork; (**D**) pectin/0.75 g cork/0.25 g coffee grounds; (**E**) pectin/0.75 g cork/0.25 g walnut shells (scale bar = 100 µm).

The surface of each film is presented in Figure 4. The SEM micrographs showed a homogeneous and smooth surface for the pectin film. The composite films with the addition of cork and the film with cork and walnut shells presented a similar surface, although they were more heterogeneous and rougher than the pectin film. The film incorporated with coffee grounds was the film that presented a more irregular and rougher surface, as was previously shown in Figure 1D. Although the particle size is the same, the obtained results may be related to the sphericity of each particle and the fact that most of the soluble fraction of the coffee had already been used in coffee brewing.

### 3.5. Biodegradation Properties

#### 3.5.1. Seawater

**Figure 5:**
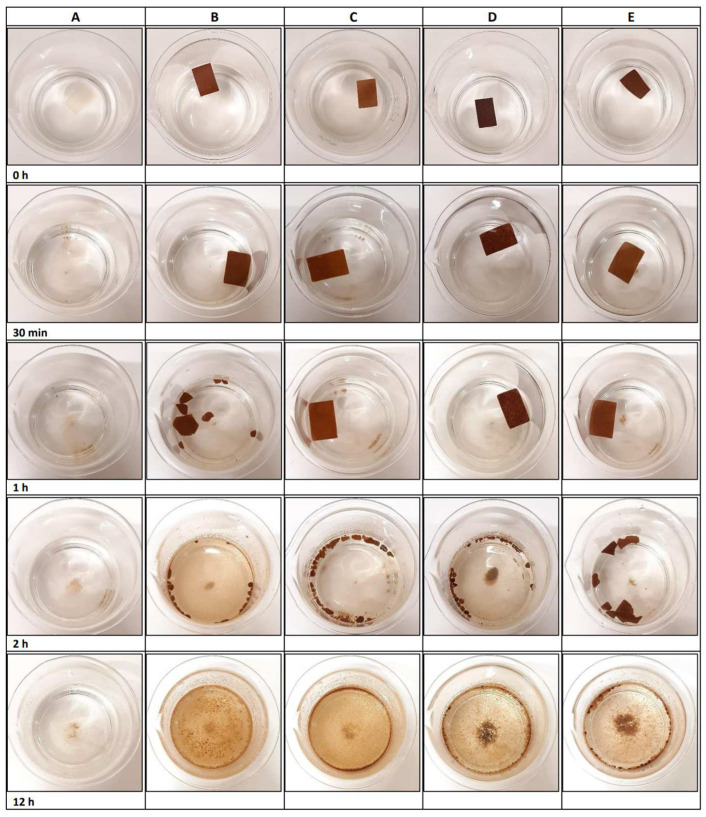
Biodegradation test in seawater: (**A**) pectin; (**B**) pectin/1 g cork; (**C**) pectin/0.75 g cork; (**D**) pectin/0.75 g cork/0.25 g coffee grounds; (**E**) pectin/0.75 g cork/0.25 g walnut shells.

Figure 5 shows the results of the biodegradation test in seawater. After the first 30 min, the samples kept their initial appearance. After the first hour of agitation in seawater, it was possible to verify that the pectin film and the pectin composite film with 1 g of cork started to be fragmented into pieces. For the control film, it was also possible to observe the clouding of the water, possibly caused by the release of pectin. After 2 h, all films were already fragmented into smaller pieces, and after 12 h, it was possible to verify that all the films were quite fragmented, losing their initial rectangle shape and therefore most of their initial structure.

There are several steps in the biodegradation process of the films in seawater. The first step is film swelling. Both film swelling and film solubility have a direct impact on the film’s water resistance properties, especially under wet conditions [38]. Nevertheless, several factors affect the rate of biodegradation of the film. These factors include the ratio of seawater to film, swelling, the presence of microorganisms in seawater, movement of the seawater, and oxygenation [35,62,63]. Nakayama et al. [64] tested the biodegradation of aliphatic polyesters in seawater and demonstrated that several factors contribute to faster biodegradation in seawater, including wave-based physicochemical effects, sunlight, and inorganic salts.

#### 3.5.2. Soil

**Figure 6:**
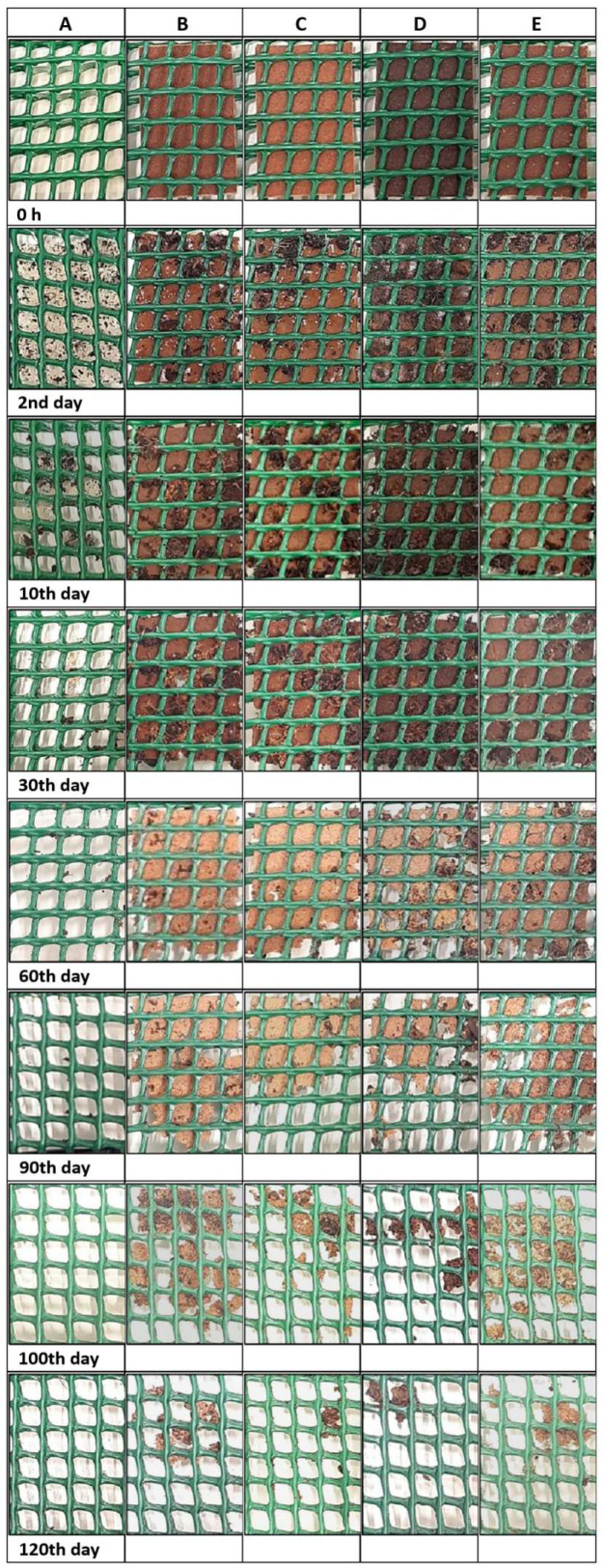
Biodegradation test in soil: (**A**) pectin; (**B**) pectin/1 g cork; (**C**) pectin/0.75 g cork; (**D**) pectin/0.75 g cork/0.25 g coffee grounds; (**E**) pectin/0.75 g cork/0.25 g walnut shells.

After 48 h in soil, the films showed some water absorption, but their structure did not present any modification (Figure 6). After 10 days, the pectin film started to show some changes in its structure, which might be related to the film’s lesser thickness and high solubility in water. After 30 days, the pectin film had practically lost its structure, while the other films started to show some small modifications. After 60, 90, and 100 days, the films with the incorporation of cork, cork with coffee grounds, or cork with walnut shells showed gradual signs of degradation in the soil with time. After 120 days, all films showed losses of about 90% in their structure. This is the value for packaging to be considered biodegradable by biological action within a period of 6 months [65].

The degradation observed is associated with the activity of microorganisms found in the soil and the specific properties of the soil. As stated by Shah et al. [66], the typical microorganisms found in the soil that contribute to biodegradation are bacteria and fungi, including *Acidovorax facilis*, *Aspergillus fumigatus*, *Comamonas* sp., *Pseudomonas lemoignei*, and *Variovorax paradoxus*. Furthermore, the presence of organic matter and the level of phosphorus in the soil play a significant role in increasing the abundance of fungi, which, in turn, contribute to the process of biodegradation [67]. The degradation process can initially occur due to a range of physical and biological forces, including temperature fluctuations, freezing and thawing, or moisture changes, leading to mechanical harm such as the fracturing of polymeric materials. The films experienced initial breakdown due to the presence of water in our research. Subsequently, depolymerization took place during the degradation process, where the extracellular enzymes from microorganisms broke down the polymer. This process resulted in the formation of shorter chains or smaller molecules, such as oligomers, dimers, and monomers, which can pass through the semipermeable outer bacterial membranes. These shorter molecules were further transformed into biodegradation end-products, including CO_2_, H_2_O, and biomass [66,68].

In general, biodegradation in seawater was obtained in a very short period, with an average degradation rate of 8.25% per hour. On the other hand, biodegradation in soil was a slower process, with an average degradation rate of 0.75% per day (in both tests, the control film degraded at a higher rate than the composite films). The degradation of the cork, coffee-grounds and walnut-shell fractions was not monitored because they are all natural products that are expected to be biodegradable (though this is known to occur at a much lower rate than pectin). Plastic- or paper-packaging degradation is an extremely gradual process, and it may require numerous years for these materials to completely decompose, as this is contingent upon the specific type of plastic or paper and the prevailing environmental conditions [69,70]. Chamas et al. [71] have reported that low-density polyethylene (LDPE) bags are projected to decompose by 50% within approximately 4.6 years when buried inland, and within around 3.4 years in marine environments. Another study conducted by Olaosebikan et al. [72] revealed that after being exposed to soil for a period of 10–12 weeks, brown newspapers began to degrade, leaving only fragments behind, while plastic bags had undergone thinning and became transparent.

### 3.6. Genotoxicity

All results of the Ames MPF assay for the tested films were negative. This indicates the absence of any DNA-reactive mutagenic substances, which can be a potential health concern (Table 4). In a recent study, Mayrhofer et al. [73] assessed recycled plastic samples including, PE, PP, PS, and PET, to determine if DNA-reactive mutagenic substances were present. DNA-reactive mutagenic substances were not detected in recycled PET, but in other types of recycled plastics (PE, PP, PS), they were detected in 51 samples from a total of 119 recycled plastic samples.

## 4. Conclusions

Bio-based materials from food by-products have the potential to provide sustainable alternatives to plastic (packaging). The present study focused on a total of three previously underused side streams and was able to demonstrate the feasibility of processing them into flexible films. In more detail, it could be shown that the incorporation of cork, cork with coffee grounds, or cork with walnut shells is suitable for the development of pectin composite films with improved properties, such as tensile strength, color, and antioxidant properties, although with more heterogeneous surfaces. This study also revealed that the developed films do not contain DNA-reactive substances and that they are biodegradable in soil and seawater. These positive properties could subsequently make the developed films an interesting eco-friendly food packaging solution that contributes to the valorization of organic waste and by-products, thus promoting the circular economy and reducing the environmental impact of plastic materials. However, further research and development is needed to achieve this goal. This includes not only research into the materials (including the films’ shelf-life and adding other bioactive substances such as gingerols [74]) and the production process but also the applicability of the materials for certain types of food (e.g., dried fruit and nuts) and their actual sustainability on an ecological, economic, and social level.

## Figures and Tables

**Table 1:** Extracted sample weight and sample concentration in acetic acid for each sample.

Sample Code	A	B	C	D	E
Sample weight (g)	0.0424	0.2663	0.2739	0.2383	0.3000
Dried sample residue (g)	0	0.1313	0.1229	0.114	0.1470
Sample in acetic acid (g)	0.0424	0.135	0.151	0.1243	0.1530
Percentage of sample extracted by acetic acid (%)	100	51	55	52	51
Sample concentration in solvent (mg/mL)	14.3	64.3	71.7	59.3	72.4

**Table 2:** Extracted sample weight and sample concentration in ethanol for each sample.

Sample Code	A	B	C	D	E
Sample weight (g)	0.0379	0.3002	0.2413	0.2378	0.2794
Dried sample residue (g)	0.0338	0.158	0.1197	0.1202	0.1498
Sample in ethanol (g)	0.0424	0.1350	0.1510	0.1243	0.1530
Percentage of sample extracted by ethanol (%)	11%	47%	50%	49%	46%
Sample concentration in solvent (mg/mL)	1.48	57.15	47.65	46.18	51.67

**Table 3:** Physico-mechanical and antioxidant properties of the developed films (A—pectin; B—pectin/1 g cork; C—pectin/0.75 g cork; D—pectin/0.75 g cork/0.25 g coffee grounds; E—pectin/0.75 g cork/0.25 g walnut shells).

	Film A	Film B	Film C	Film D	Film E
Thickness (mm)	0.074 ± 0.003 ^a^	0.552 ± 0.021 ^b^	0.487 ± 0.014 ^c^	0.572 ± 0.014 ^b^	0.553 ± 0.028 ^b^
Color					
L*	91.86 ± 0.18 ^a^	33.32 ± 0.51 ^b^	33.93 ± 0.84 ^b^	25.30 ± 0.78 ^c^	30.52 ± 1.14 ^b^
a*	1.20 ± 0.12 ^a^	14.76 ± 0.39 ^b^	14.73 ± 0.40 ^b^	6.19 ± 0.38 ^c^	12.60 ± 1.43 ^b^
b*	15.62 ± 0.71 ^a^	16.39 ± 0.79 ^a^	16.39 ± 0.82 ^a^	6.55 ± 0.53 ^b^	13.77 ± 1.67 ^a^
Opacity (%)	12.00 ± 0.40 ^a^	99.50 ± 0.54 ^b,c^	99.70 ± 0.62 ^b,c^	99.80 ± 0.54 ^c^	96.70 ± 1.71 ^b,d^
Water-vapor permeability (g/(m.s.Pa))	3.84 × 10^−10^ ± 1.82 × 10^−11 a^	1.75 × 10^−9^ ± 2.27 × 10^−11 b^	1.88 × 10^−9^ ± 4.00 × 10^−11 b^	4.52 × 10^−9^ ± 1.60 × 10^−10 c^	5.87 × 10^−9^ ± 1.82 × 10^−10 d^
Water solubility (%)	100.00 ± 0.00 ^a^	55.70 ± 2.50 ^b^	70.10 ± 2.60 ^c^	51.90 ± 6.70 ^b,c^	56.20 ± 6.20 ^b,c^
Moisture content (%)	12.80 ± 7.20 ^a^	49.50 ± 3.60 ^b^	49.60 ± 4.50 ^b^	48.60 ± 3.40 ^b^	45.90 ± 0.20 ^b^
Elongation at break (%)	2.50 ± 0.5 ^a^	36.30 ± 3.25 ^b^	32.24 ± 1.88 ^b^	7.50 ± 0.76 ^c^	2.50 ± 0.19 ^a^
Young’s modulus (MPa)	0.99 ± 0.07 ^a^	0.16 ± 0.01 ^b^	0.15 ± 0.03 ^b,c^	0.19 ± 0.01 ^c^	0.16 ± 0.02 ^b,c^
Tensile strenght (MPa)	20.47 ± 2.55 ^a^	0.91 ± 0.19 ^b^	1.09 ± 0.08 ^b^	0.99 ± 0.09 ^b^	1.05 ± 0.06 ^b^
TPC (mg Gallic acid/g)DPPH (mg Trolox/g)ABTS (mg Trolox/g)	0.96 ± 0 ^a^ 0.31 ± 0 ^a^ 0.99 ± 0.01 ^a^	3.76 ± 0.02 ^b^ 5.57 ± 0.21 ^b,c^ 8.73 ± 0.04 ^b^	3.17 ± 0.01 ^c^ 4.49 ± 0.06 ^d^ 6.34 ± 0.40 ^c^	3.88 ± 0.02 ^d^ 5.60 ± 0.10 ^b^ 6.80 ± 0.10 ^d^	4.24 ± 0.02 ^e^ 5.35 ± 0.05 ^c^ 9.08 ± 0.30 ^b^

Different superscript letters indicate significant differences (*p* < 0.05).

**Table 4:** Ames MPF: A—pectin; B—pectin/1 g cork; C—pectin/0.75 g cork; D—pectin/0.75 g cork/0.25 g coffee grounds; E—pectin/0.75 g cork/0.25 g walnut shells. x_A, extraction in acetic acid; x_E, extraction in ethanol. *Salmonella* Typhimurium TA98 (TA98) and *Salmonella* Typhimurium TA100 (TA100). Phenobarbital/β-naphtoflavone-induced rat liver S9 (S9). (-) negative.

Sample	TA98	TA98 + S9	TA100	TA100 + S9
A_A	-	-	-	-
B_A	-	-	-	-
C_A	-	-	-	-
D_A	-	-	-	-
E_A	-	-	-	-
Acetic Acid	-	-	-	-
A_E	-	-	-	-
B_E	-	-	-	-
C_E	-	-	-	-
D_E	-	-	-	-
E_E	-	-	-	-
95% Ethanol	-	-	-	-

## Data Availability

Data are contained within the article.
